# Caffeic acid ethanolamide prevents cardiac dysfunction through sirtuin dependent cardiac bioenergetics preservation

**DOI:** 10.1186/s12929-015-0188-1

**Published:** 2015-09-22

**Authors:** Shih-Yi Lee, Hui-Chun Ku, Yueh-Hsiung Kuo, Kai-Chien Yang, Ping-Chen Tu, His-Lin Chiu, Ming-Jai Su

**Affiliations:** Institute of Pharmacology, College of Medicine, National Taiwan University, No.1, Sec.1, Jen-Ai Road, Taipei, 10051 Taiwan; Division of Pulmonary and Critical Care Medicine, Mackay Memorial Hospital, Taipei, Taiwan; Mackay Junior College of Medicine, Nursing, and Management, Taipei, Taiwan; Department of Chinese Pharmaceutical Sciences and Chinese Medicine Resources, China Medical University, Taichung, Taiwan; Department of Biotechnology, Asia University, Taichung, Taiwan; Department of Chemistry, National Taiwan University, Taipei, Taiwan

**Keywords:** Bioenergetics, Caffeic acid, Heart failure, Sirtuin

## Abstract

**Background:**

Cardiac oxidative stress, bioenergetics and catecholamine play major roles in heart failure progression. However, the relationships between these three dominant heart failure factors are not fully elucidated. Caffeic acid ethanolamide (CAEA), a synthesized derivative from caffeic acid that exerted antioxidative properties, was thus applied in this study to explore its effects on the pathogenesis of heart failure.

**Results:**

In vitro studies in HL-1 cells exposed to isoproterenol showed an increase in cellular and mitochondria oxidative stress. Two-week isoproterenol injections into mice resulted in ventricular hypertrophy, myocardial fibrosis, elevated lipid peroxidation, cardiac adenosine triphosphate and left ventricular ejection fraction decline, suggesting oxidative stress and bioenergetics changes in catecholamine-induced heart failure. CAEA restored oxygen consumption rates and adenosine triphosphate contents. In addition, CAEA alleviated isoproterenol-induced cardiac remodeling, cardiac oxidative stress, cardiac bioenergetics and function insufficiency in mice. CAEA treatment recovered sirtuin 1 and sirtuin 3 activity, and attenuated the changes of proteins, including manganese superoxide dismutase and hypoxia-inducible factor 1-α, which are the most likely mechanisms responsible for the alleviation of isoproterenol-caused cardiac injury

**Conclusion:**

CAEA prevents catecholamine-induced cardiac damage and is therefore a possible new therapeutic approach for preventing heart failure progression.

## Background

Heart failure (HF) remains a major cause of death in developed nations [1]. It is a complex and multi-causal syndrome characterized by cardiac dysfunction [2–6]. Evidence has shown that catecholamine, oxidative stress and bioenergetic insufficiency contribute to the pathogenesis of HF [7–13]. The increase in sympathetic tone in HF is supposed to compensate for cardiac dysfunction; however, a previous study found that the patients with higher plasma catecholamine concentrations had poorer outcomes [14]. A synthetic catecholamine, isoproterenol (ISO), has also been widely used to induce oxidative stress HF, displaying cardiac remodeling, dysfunction, and bioenergetics insufficiency [15–17]. These observations imply that catecholamine released to counterbalance the cardiac dysfunction could further result in myocardial oxidative injury and bioenergetics impairment in HF.

Mitochondria are responsible for oxidative phosphorylation. Adenosine triphosphate (ATP) is produced from the electron transport chain (ETC) which supplies energy for well-perfused hearts [12, 18, 19]. On the other hand, reactive oxygen species (ROS) leaking from impaired ETC in failing myocardium contributes to mitochondrial and cellular oxidative stress, further deteriorating cardiac bioenergetics [9, 10, 13, 18, 20–29]. Accordingly, amelioration of mitochondrial oxidative stress has been considered as a possible resolution to heart failure [23, 26]. Agents that correct impaired ETC can reduce ROS leakage from mitochondria [30, 31]. Modulation of the cellular oxidative alternation is another possible therapeutic modality [31, 32] and attenuating mitochondrial oxidative stress is yet another [33].

Sirtuins (SIRTs) are family of class III histone deacetylases, which require NAD^+^ to deacetylate histone and nonhistone lysines [34]. Mammals contain seven sirtuins, SIRT1–7 [35]. SIRT1 and SIRT3 are highly expressed in the nucleus/cytoplasm and mitochondria of the heart, respectively [34–37]. It has been shown that sirtuin 1 (SIRT1) is downregulated in patients with heart failure, and that there is an increase in sirtuin 1 reducing oxidative stress-mediated cardiac reperfusion injury [38, 39]. Meanwhile, sirtuin 3 (SIRT3) has been demonstrated to regulate cardiac energy status and mitochondria tolerance to ischemia-reperfusion injury by deacetylating specific mitochondrial proteins [40, 41]. Therefore, SIRT 1 and SIRT 3 are potential targets for managing catecholamine inducing oxidative stress and bioenergetic insufficiency, thus preventing the progression of HF.

Caffeic acid, a natural phenolic constituent, has antioxidative properties [42, 43]. Its cardiovascular protection has been demonstrated through its free radical scavenging effect [44–50]. However, the exact mechanisms underlying caffeic acid-induced cardio-protection and its therapeutic potential on HF remain unknown. In addition, our preliminary data represented that a new caffeic acid derivate, caffeic acid ethanolamide (CAEA), exerted cardioprotective effects, which was superior to caffeic acid (data will show later). We aimed in our present study to evaluate the effects of CAEA on catecholamine-induced HF, and the involved mechanisms.

## Methods

### Experimental animals and ethics statement

Eight-week-old male C57BL/6 mice were purchased from the National Laboratory Animal Center of Taiwan. The research was performed according to the Guide for the Care and Use of Laboratory Animals published by the US National Institutes of Health (NIH publication no. 85–23, revised 1996), and was approved by the Institutional Animal Care and Use Committee of the National Taiwan University, Taiwan. ISO (Sigma-Aldrich, St. Louis, MO) 16 mg/kg once daily was subcutaneously injected for 14 days. The control groups received the same volume of isotonic saline. CAEA suspended in isotonic saline was administered subcutaneously as a dose of 1 mg/kg/day after ISO injection. In some experiments, nicotinamide (20 mg/kg/day), a sirtuin inhibitor, was also injected subcutaneously to investigate the mechanism of CAEA.

### Caffeic acid ethanolamide preparation

**Fig. 1:**
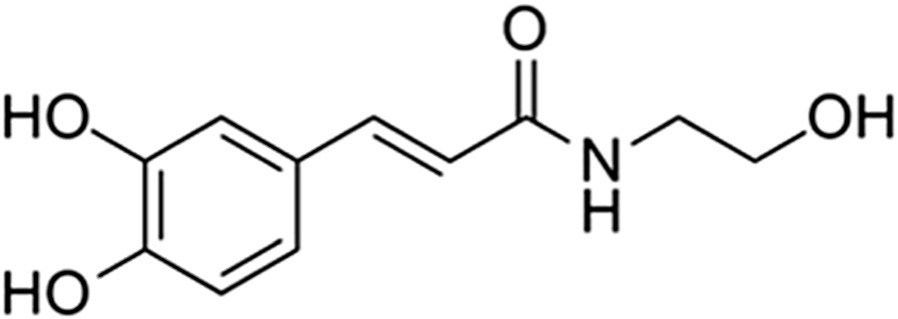
Structure of caffeic acid ethanolamide

CAEA is synthesized in the laboratory of YH Kuo (Fig. 1). CAEA was produced from caffeic acid (100 mg, 0.56 mmole) dissolved in 1 mL N,N-dimethylformamide and 80 μL triethylamine in a two-necked bottle. The solution was then added into 5 mL dichloromethane containing 41 μL (1.2 eq) ethanolamine, and 298 mg (1.2 eq) (Benzotriazol-1-yloxy)tris- (dimethylamino)phosphonium hexafluorophosphate to react for 30 min in an ice bath, followed by reacting at room temperature for 2 h. After the reaction, dichloromethane was removed with low negative pressure. The residue was then added into water, and then extracted by ethyl acetate. The organic phase was then collected, washed with 3 N HCl, 10 % NaHCO_3_ and water, and then dried with anhydrous sodium sulfate. After filtration, condensation, and column chromatography, the final product- caffeic acid ethanolamide was obtained.

### Cardiac function assessment

After 14 days of drug administration, small animal ultrasound imaging system (S-Sharp Corporation, Taipei, Taiwan) was used for echocardiography measurements. Transthoracic echocardiography was performed 12 h after the last drug injection. Mice were anesthetized by 2 % isoflurane mixed with 1 L/min O_2_ in the induction chamber, while the continuous application of anesthesia was dropped to 1 % isoflurane. Cardiac function was calculated, in duplicate, in M-mode images from the parasternal long axis by using the leading-edge technique defined by the American Society of Echocardiography. Left ventricle ejection fraction (EF) is an indicator for the determination of cardiac function.

### Cardiac histology

After the echocardiogram was recorded, the heart was excised and perfused with PBS. The weight of heart was measured, and the heart to body weight (HW/BW) ratio was calculated. The hearts were fixed in 4 % paraformaldehyde, embedded in paraffin, and sectioned horizontally in 4 μm slices. Masson’s trichrome stain and Sirius red stain were performed for fibrosis analysis.

### Cardiac protein extraction

Left ventricles were homogenized as described previously [51]. Briefly, left ventricles were homogenized in tissues protein extraction buffer (Thermo Fisher Scientific Inc., IL, USA) containing cocktail proteases and phosphatase inhibitors (Sigma, St. Louis, MO, USA). The supernatant of the tissue homogenate was collected after centrifugation (800 × g, 10 min at 4 °C) and was defined as total cardiac protein. Protein concentrations were determined by a BCA protein assay kit (Thermo Fisher Scientific Inc., IL, USA).

### ATP content determination

Mouse ventricular tissue lysate was prepared for measuring cardiac ATP content, which was measured by an ELISA kit (Biovision, CA, USA). To detect lactate content, the ATP reaction mix was mixed well with tissue lysate in each well at room temperature protected from light for 30 min, and the fluorescence signals were detected by excitation wavelength of 535 nm and an emission wavelength of 587 nm with a microplate spectrophotometer.

### Western blotting

Cardiac protein samples were analyzed for manganese superoxide dismutase **(**MnSOD), c-Jun N-terminal kinase (JNK), phospho-JNK (p-JNK) and hypoxia-inducible factor 1-α (HIF-1α) expression (Cell Signaling, MA, USA), and glyceraldehyde 3-phosphate dehydrogenase (GAPDH) (Santa Cruz Biotechnology, CA, USA). The methods were described in our previous study [51].

### Sirtuin activity detection

Mouse ventricular tissue lysates were prepared for the measurement of SIRT1 and SIRT3 activities, which were measured by kits (Cayman Chemicals, MI, USA). p53 sequence, as the substrate for sirtuin deacetylation, was mixed with tissue lysate in a 96 well microplate, and was then shaken at 25 °C for 45 min. Fluorescence signals were detected by an excitation wavelength of 360 nm and an emission wavelength of 450 nm with a microplate spectrophotometer.

### Lactate content and the ratio of oxidized and reduced forms of nicotinamide adenine dinucleotides (NAD^+^/NADH ratio) detection

Mouse ventricular tissue lysate was prepared for the measurement of lactate content and NAD^+^/NADH ratio, which were both measured by ELISA kits (Biovision, CA, USA). To detect lactate content, the lactate reaction mix was added to each well along with tissue lysate at room temperature away from light for 30 min, and then read at an optical density of 570 nm. In addition, after centrifuging the samples at 14,000 rpm for 5 min, the supernatant of the heart tissue was transferred into a new tube. To detect total NAD, the supernatant was mixed with an NADH developer in each well of a 96 well microplate at room temperature for 5 min, and then the color was read at an OD of 450 nm. To detect NADH, we heated the supernatant to 60 °C for 30 min to decompose NAD^+^, and then we followed the steps of the reaction mentioned above. After the standard curve was prepared, the NAD+/NADH ratio was obtained from the total NAD and NADH detected, which is equal to the (total NAD - NADH)/NADH ratio.

### Lipid peroxidation determination

Cardiac oxidative stress was represented by lipid peroxidation of mouse ventricular tissue, and determined by a kit (Cayman Chemicals, MI, USA). Briefly, after centrifuging the samples at 1,600 g at 4 °C for 10 min, the supernatant was mixed with sodium dodecyl sulfate solution along with the color reagent in tubes, and then put them into boiling water for 1 h, followed by incubating them on ice for 10 min. After centrifuging the samples at 1,600 g at 4 °C for 10 min, we read the fluorescence signals at the excitation wavelength of 350–360 nm and an emission wavelength of 450–465 nm by using a microplate spectrophotometer.

### Cell culture

HL-1 cells, a cardiac muscle cell line that contracts and retains phenotypic characteristics of the adult cardiomyocyte, were obtained from Dr. William C. Claycomb (Louisiana State University Health Sciences Center, New Orleans, LA). Cells were cultured in Claycomb medium supplemented with 10 % FBS (Gibco, Scotland, UK), 2 mM L-glutamine (Gibco, Scotland, UK), 0.1 mM norepinephrine, and antibiotics (100 μg/ml penicillin and 100 μg/ml streptomycin) at 37 °C under a 5 % CO_2_ − 95 % air atmosphere. The HL-1 cells were used for experimentation after reaching 80 % confluency. ISO was added to induce stress for 24 h. CAEA (1 μM) was pre-incubated 1 h before ISO treatment.

### Intracellular free radical determination

Intracellular ROS and mitochondria superoxide generation was detected in cardiomyocytes by labeling with fluorescence dye 5-(and-6)-chloromethyl-2′,7′- dichlorodihydrofluorescein diacetate and MitoSOX™, respectively. By using fluorescence microscopy, intracellular ROS level was monitored at 488 nm excitation and 515 nm emission, and mitochondria superoxide generation was monitored at 510 nm excitation and 580 nm emission, respectively. Fluorescence intensity was calculated by averaging fluorescence intensity of numerous outlined cells using ImageQuant (Molecular Dynamics, Inc., Sunnyvale, CA, USA).

### Rate of oxygen consumption detection

To assess the function of the cellular electron transport chain, oxygen consumption rate (OCR) was estimated by a kit (Luxcel Biosciences Ltd., Cork, Ireland). MitoXpress^R^ Xtra was added to each well containing cells after treatment. The dual-read signal was recorded continuously right after mineral oil sealing. Since the detection dye is quenched by O_2_ through molecular collision, the fluorescence signal is inversely proportional to the amount of extracellular O_2_ in the sample. Rates of oxygen consumption were determined from the changes in the fluorescence signals over time. The slope between linear regression lifetime of fluorescence and detection period was calculated as OCR. The values of OCR were normalized to protein content.

### pH level determination

pH values were measured in cell culture, by adding a pH-sensitive fluorescence dye (Invitrogen, NY, USA). The fluorescence signal is proportionally increased during the lowering of the pH value, and detected by excitation wavelength of 560 nm and an emission wavelength of 585 nm.

### Glycolysis detection

Cellular glycolysis was measured by a kit (Luxcel Biosciences Ltd., Cork, Ireland). After several washes, pH-Xtra™ was added to the cells, and the fluorescence signal was recorded in a continuous dual-read manner. The values of the glycolysis rate were normalized to protein content.

### Statistical analysis

All values were represented as means ± SE. The results were analyzed using ANOVA followed by Bonferroni's post hoc tests. *P* <0.05 was considered as a significant difference.

## Results

### CAEA prevents isoproterenol caused myocardial remodeling

**Fig. 2:**
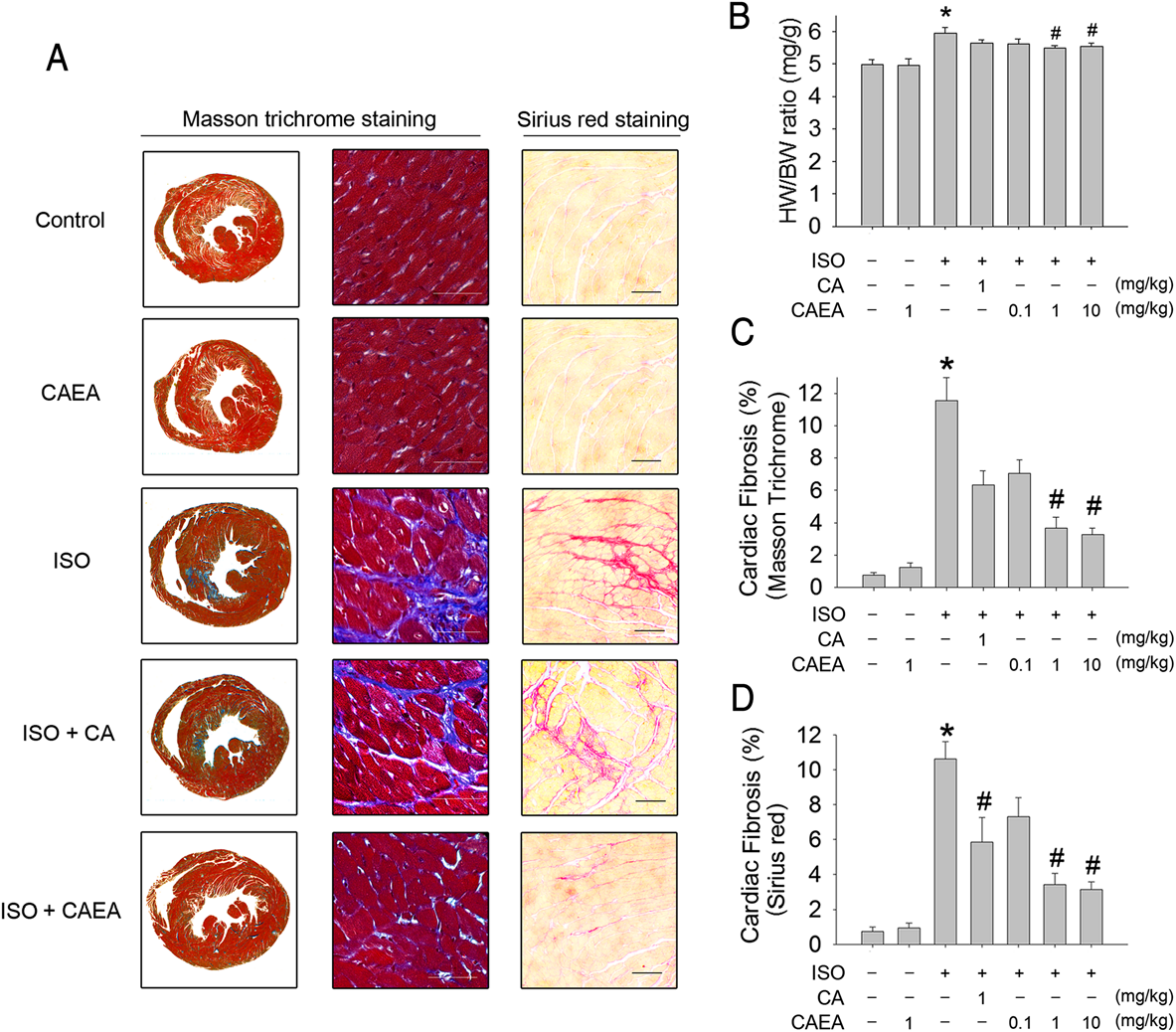
Effects of caffeic acid ethanolamide (CAEA) on cardiac remodeling in mice subjected to two weeks subcutaneous isoproterenol (ISO) injections. **a** Representative histological sections. **b** Heart weight to body weight (HW/BW) ratio. **c** Cardiac fibrosis in blue assessed by Masson’s trichrome staining **d** Quantification of cardiac fibrosis in pink assessed by Sirius red staining (bar = 100 μm). *n* = 9, *P <0.05 versus control, ^#^ *P* <0.05 versus ISO

CAEA (1 mg/kg) alone had no significant impact on cardiac morphology and histology (Fig. 2). Conversely, the two-week ISO induced cardiac remodeling- ventricular hypertrophy and myocardial fibrosis was alleviated by CAEA (1 mg/kg). The cardio-protective effects of CAEA were superior to caffeic acid (1 mg/kg) in terms of isoproterenol-induced cardiac remodeling (Fig. 2). The ratio of heart weight to body weight was considerably reduced from 5.95 mg/g in ISO-treated mice (ISO group) to 5.49 mg/g in mice subjected to CAEA and ISO (ISO + CAEA group), while that in the vehicle-treated mice (control group) was 4.95 mg/g (Fig. 2ab). Meanwhile, cardiac fibrosis was significantly attenuated from 11.02 % in the ISO group to 3.67 % in the ISO + CAEA group, when compared with 0.77 % in the control group (Fig. 2cd).

### CAEA alleviates isoproterenol induced cardiac dysfunction and bioenergetic insufficiency

**Fig. 3:**
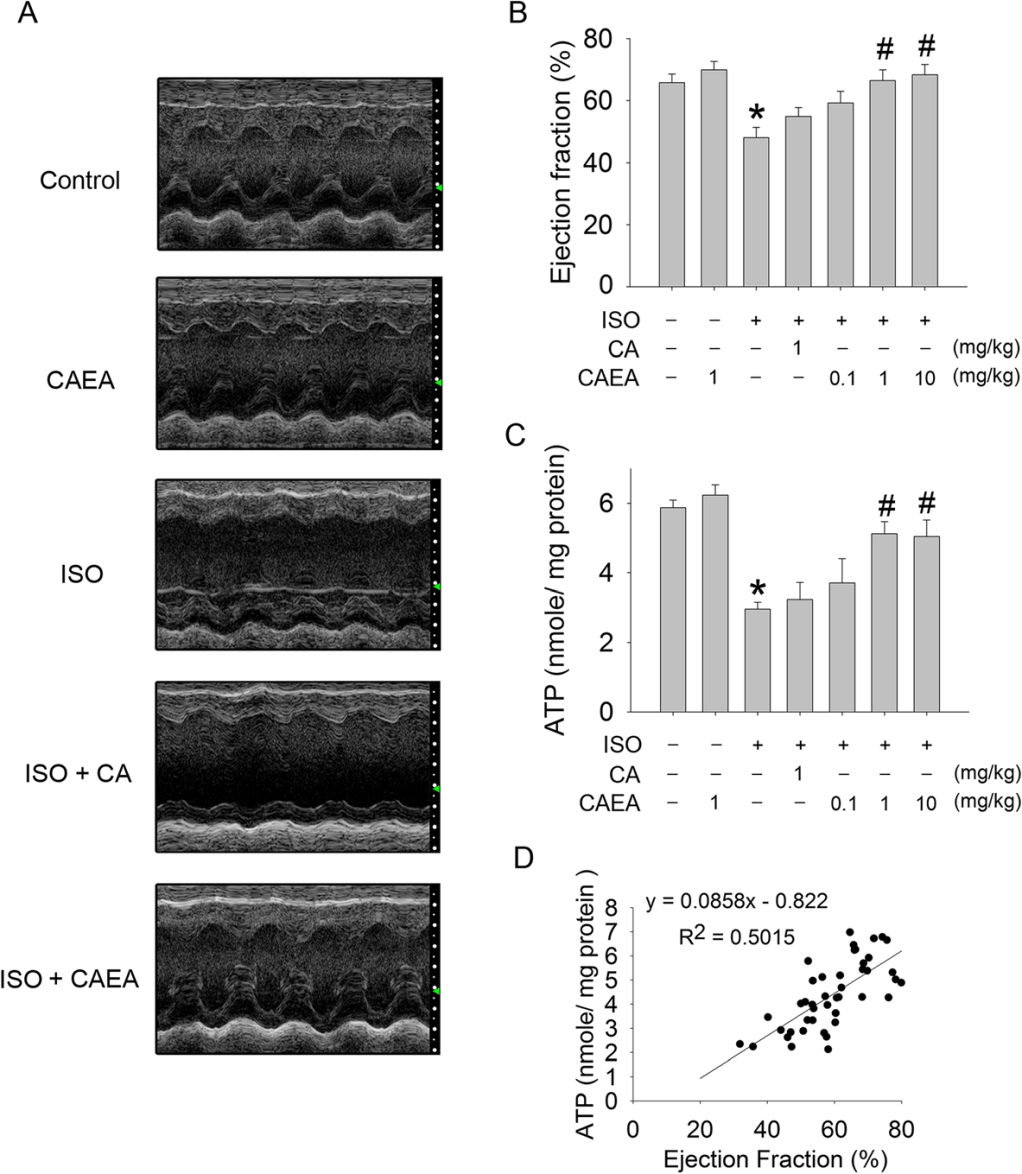
Effects of caffeic acid ethanolamide (CAEA) on cardiac function and cardiac energy in mice subjected to two weeks subcutaneous isoproterenol (ISO) injections. **a** Representative M-mode echocardiogram. **b** Quantification of left ventricular ejection fraction. **c** Quantification of ATP contents. **d** The correlation between left ventricular ejection fraction and ATP contents (R^2^: coefficient of determination). *n* = 9, *P <0.05 versus control, ^#^ *P* <0.05 versus ISO

CAEA (1 mg/kg) alone did not change left ventricle ejection fraction (LVEF) and cardiac ATP (Fig. 3abc). LVEF declined from 65.8 % in the control group to 48.2 % in the ISO group (Fig. 3ab). The decline of LVEF in the ISO group was significantly attenuated to 66.4 % in the ISO + CAEA group (Fig. 3ab). In the meantime, the drop of cardiac ATP in the ISO group (50.4 %) was preserved in the ISO + CAEA group (86.2 %), when compared with the control group (Fig. 3c). LVEF correlated well with cardiac ATP content in mice (Fig. 3d).

Again, CAEA showed its superiority over caffeic acid when considering isoproterenol-induced cardiac bioenergetic impairment and dysfunction. Therefore, we chose CAEA for further evaluation (Fig. 3abc).

### CAEA recovers cardiac manganese superoxide dismutase and reduces oxidative stress in isoproterenol induced heart failure

**Fig. 4:**
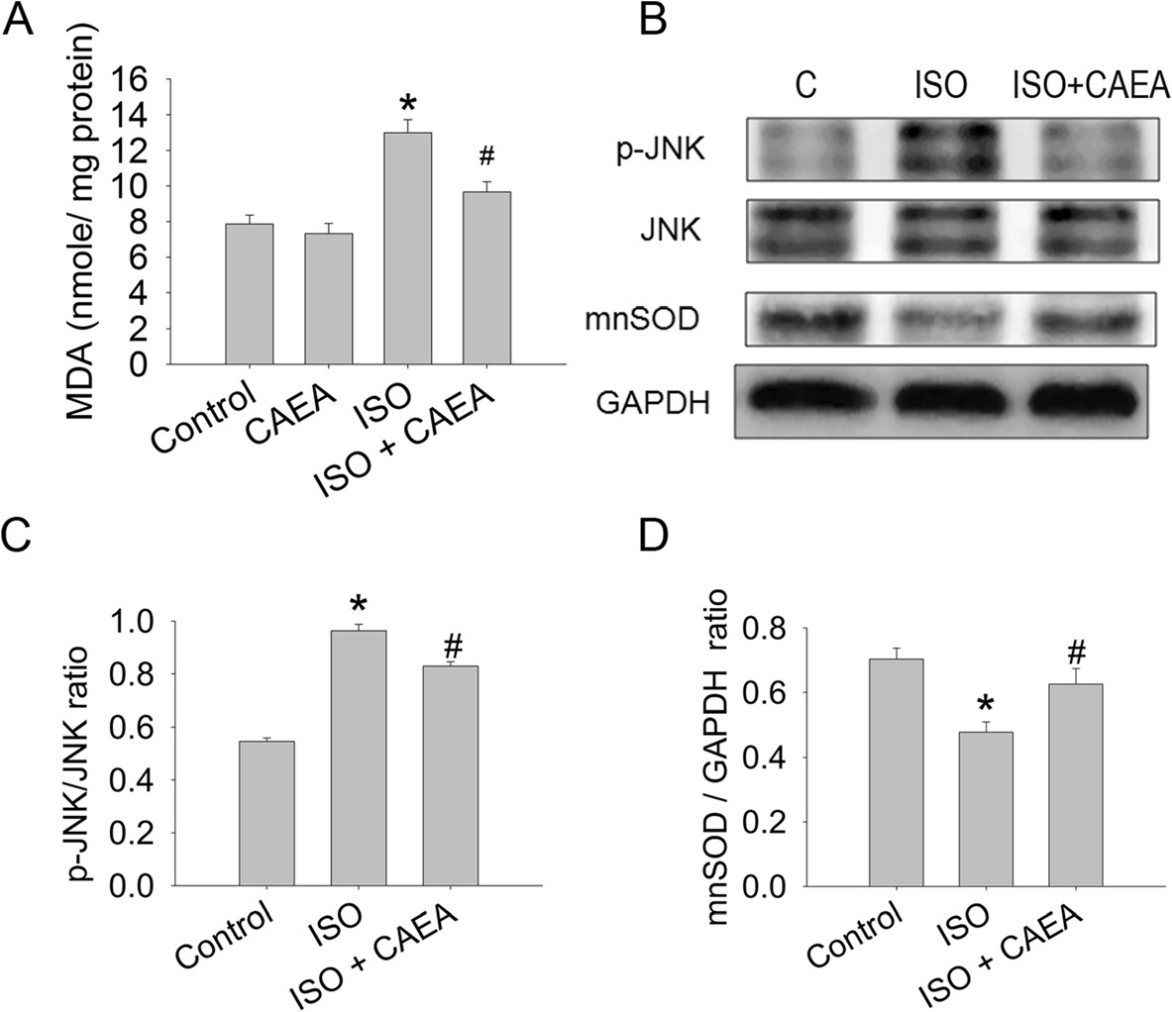
Effects of caffeic acid ethanolamide (CAEA) on cardiac oxidative stress in mice subjected to two weeks subcutaneous isoproterenol (ISO) injections. **a** Cardiac tissue lipid peroxidation determined by malondialdehyde (MDA). **b** Representative Western blot image of cardiac tissue phosphorylation of JNK, and manganese superoxide dismutase (mnSOD) expression. **c** Densitometry of cardiac tissue phosphorylation of JNK and **d** mnSOD expression. *n* = 6 ~ 9, *P <0.05 versus control, ^#^ *P* <0.05 versus ISO

ISO increased cardiac oxidative stress, which was measured as lipid peroxidation. CAEA alleviated ISO induced cardiac oxidative stress from 1.65- to 1.23-fold, when compared with control group (Fig. 4a). Cardiac protein expression was analyzed by Western blotting. CAEA ameliorated ISO induced JNK phosphorylation from 1.76- to 1.52-fold higher, compared to the control group (Fig. 4bc), while cardiac MnSOD, which was 67.9 % in ISO group, recovered to 88.9 % in the ISO + CAEA group, compared to the control group (Fig. 4bd).

### Isoproterenol increases cellular and mitochondrial oxidative stress in HL-1 cardiomyocytes, while CAEA reduces them both

**Fig. 5:**
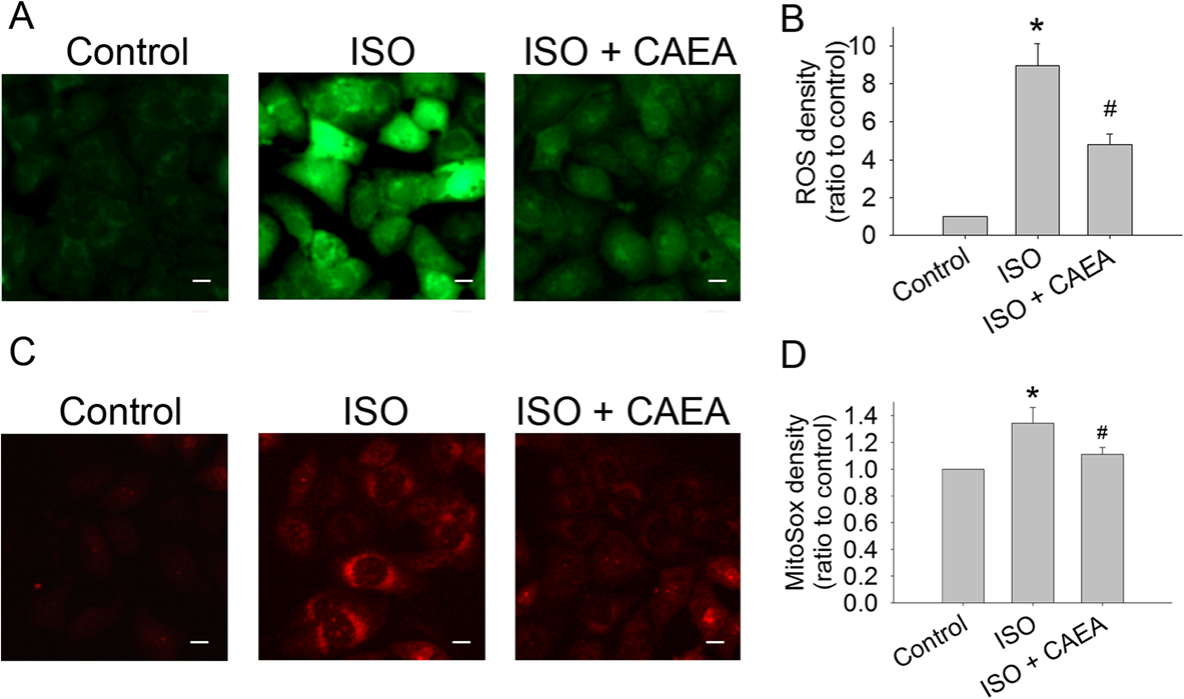
Effects of caffeic acid ethanolamide (CAEA) on oxidative stress in HL-1 cells exposed to isoproterenol (ISO). **a** Cellular reactive oxygen species (ROS) in green fluorescence (bar = 10 μm). **b** Quantification of cellular ROS. **c** Mitochondrial superoxide in red fluorescence (bar = 10 μm). **d** Quantification of mitochondrial superoxide. *n* = 4 in triplicate for each group, *P <0.05 versus control, ^#^ *P* <0.05 versus ISO

ISO induced cellular oxidative stress, which was measured by fluorescence staining in HL-1 cardiomyocytes (Fig. 5). Intracellular ROS (green fluorescence) was 8.93-fold greater in the ISO group than in the control group, and was reduced to 4.81-fold elevation in the ISO + CAEA group (Fig. 5ab). In addition, mitochondrial superoxide (red fluorescence) was 1.23-fold higher in the ISO group than in the control group, and was alleviated to 1.06-fold elevation in the ISO + CAEA group (Fig. 5cd). CAEA reduced ISO caused cellular oxidative stress and mitochondrial superoxide activity.

### CAEA preserves oxidative phosphorylation, cellular bioenergetics and cellular redox state in isoproterenol-treated HL-1 cardiomyocytes

**Fig. 6:**
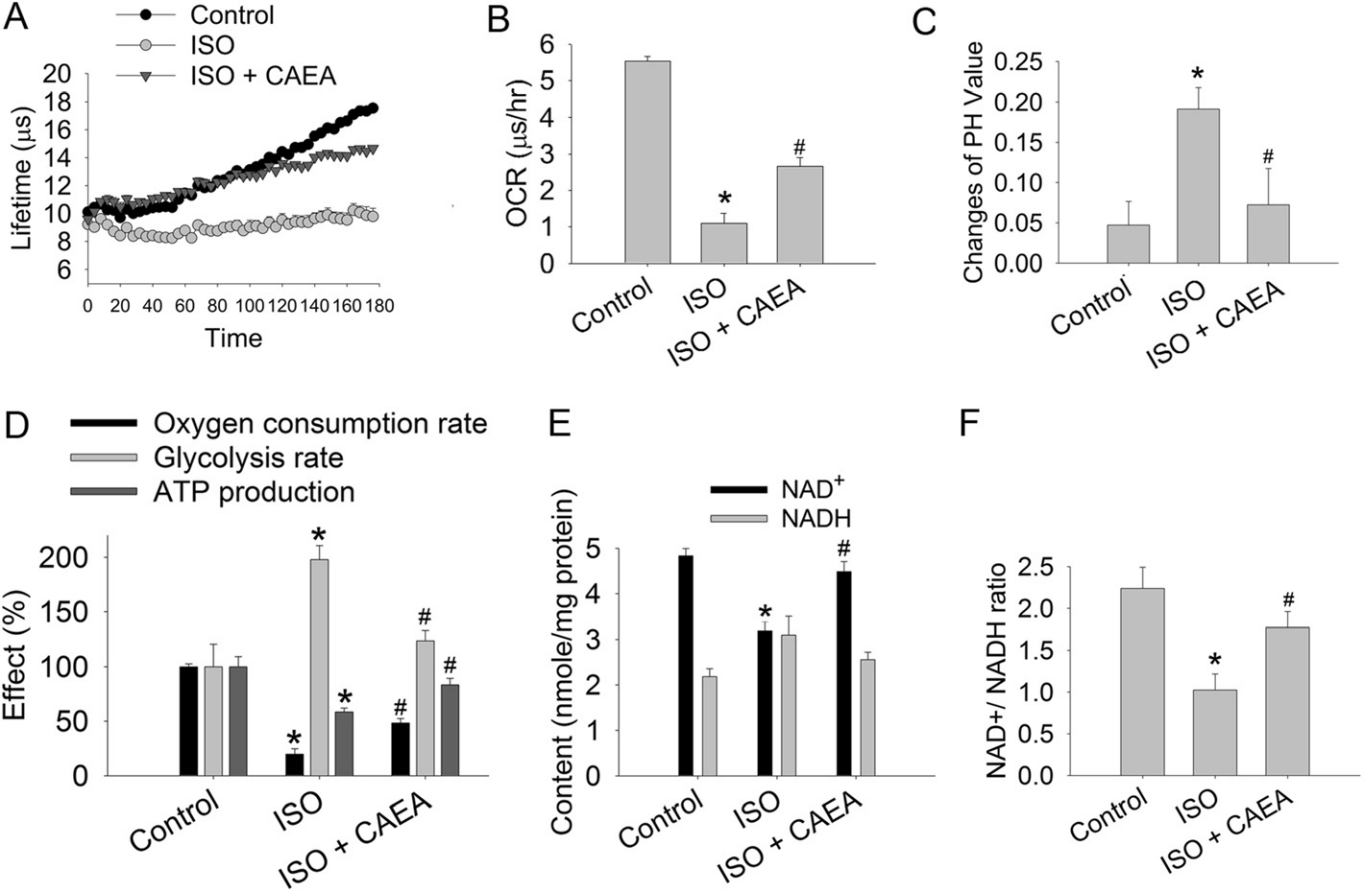
Effects of caffeic acid ethanolamide (CAEA) on cellular bioenergetics in HL-1 cells exposed to isoproterenol (ISO). **a** Lifetimes of oxygen consumption in fluorescence changes. **b** Quantification of oxygen consumption rate (OCR). **c** Quantification of intracellular pH value changes. **d** Quantification of cellular bioenergetics. **e** Quantification of cellular NAD^+^ and NADH. **f** Quantification of cellular NAD^+^/NADH. *n* = 4 in triplicate for each group, *P <0.05 versus control, ^#^ *P* <0.05 versus ISO

Cellular oxidative phosphorylation in HL-1 cardiomyocytes was represented by oxygen consumption rate (OCR). OCR declined from 5.53 μs/h in the control group to 1.09 μs/h in the ISO group, and only declined to 2.67 μs/h in the ISO + CAEA group (Fig. 6ab). The glycolysis rate was 1.9-fold higher in ISO group than control group (Fig. 6d). The ISO group showed the greatest decrease in pH values among the groups (pH 7.20) and recovered to pH 7.33 in the ISO + CAEA group (Fig. 6c). Cellular ATP in the ISO group was 58.5 % of the control group. NAD^+^ in the ISO group was 66.0 % of the control group, compared to NADH which was 1.42 fold higher than in the control group. The NAD^+^/NADH ratio (representing cellular redox state [18]) in the ISO group was 45.7 % of the control group (Fig. 6de). Taken together, ISO decreased cellular OCR, elevated the glycolysis rate and NADH, reduced cellular pH, ATP production, NAD^+^ and NAD^+^/NADH ratio (Fig. 6). Conversely, CAEA significantly reversed the effects of ISO on cellular glycolysis rate, pH, ATP production, NAD^+^, NADH and NAD^+^/NADH ratio, which were 1.23-fold, 83.3 %, 87.3 %, 92.9 %, 1.17-fold and 79.2 % of control group, respectively (Fig. 6). The preservation of cellular oxidative phosphorylation and the alleviation of glycolysis by CAEA in HL-1 cells exposed to ISO could lead to cellular ATP and redox state restoration.

### CAEA preserved cardiac bioenergetics in isoproterenol induced cardiac dysfunction is sirtuin dependent

**Fig. 7:**
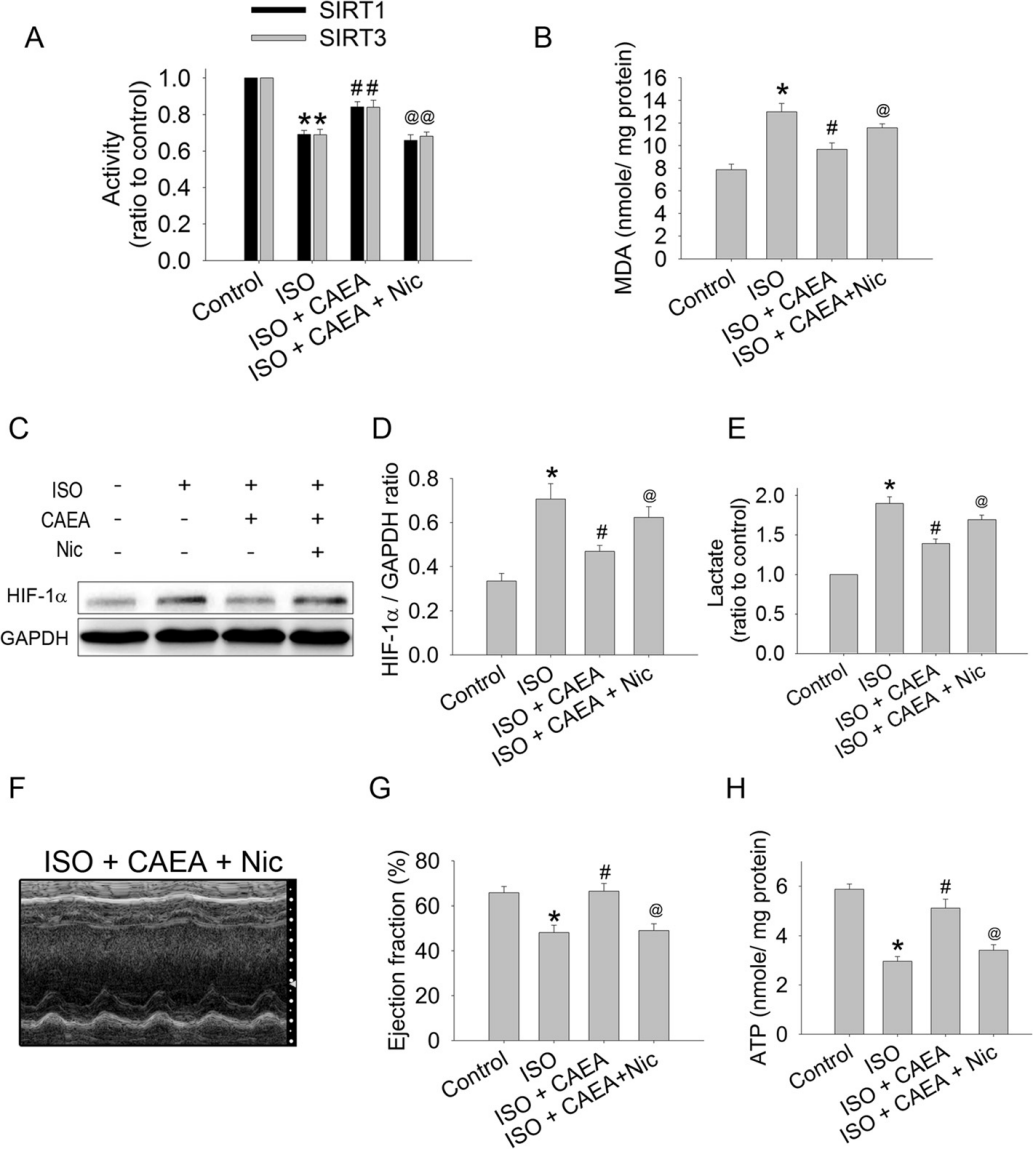
Mechanism of caffeic acid ethanolamide (CAEA) affecting cardiac oxidative stress and cardiac bioenergetics in mice subjected to two weeks subcutaneous isoproterenol (ISO) injections. **a** Quantification of cardiac sirtuin 1 (SIRT1) and sirtuin 3 (SIRT3) activity. **b** Quantification of cardiac lipid peroxidation. **c** Representative Western blot image of cardiac HIF-1α expression. **d** Densitometry of cardiac HIF-1α expression. **e** Quantification of cardiac lactate content. **f** Representative M-mode echocardiogram. **g** Quantification of left ventricular ejection fraction. **h** Quantification of cardiac ATP content. *n* = 6 ~ 9, **P <0.01 versus control, *P <0.05 versus control, ^#^ *P* <0.05 versus ISO, ^@^ *P* <0.05 versus ISO + CAEA. (Nic = nicotinamide)

CAEA did not change the SIRT1 and SIRT3 expression levels compared to the control group. However, the decline of SIRT1 and SIRT3 activity in the ISO mouse group was preserved in the ISO + CAEA group, which were elevated from 67.7 % to 82.5 % and from 68.5 % to 83.6 % of the control group, respectively (Fig. 7a). The increase in lipid peroxidation and HIF-1α expression in the ISO group (1.65-fold, 2.1-fold of control group) was significantly reduced in the ISO + CAEA group (1.23-fold, 1.4-fold of control group) (Fig. 7bcd). When sirtuin was inhibited by nicotinamide, the CAEA protective effects, including lipid peroxidation, HIF-1α expression, lactate contents, LVEF and ATP production, were all abolished (Fig. 7b ~ h). In summary, the CAEA alleviating effects of ISO induced cardiac injury were sirtuin dependent.

## Discussion

We demonstrated that CAEA alleviates cardiac remodeling and improves cardiac functions in murine ISO induced HF. CAEA recovered the SIRT1, SIRT3 activity, and MnSOD expression and downregulated HIF-1α expression, leading to a reduction in oxidative stress, preserving oxidative phosphorylation, cardiac bioenergetics, and cardiac function.

Cardiac ATP status is linked to cardiac ventricular performance [52]. Normally, two thirds of the ATP hydrolysis in cardiomyocytes is utilized for the contractile apparatus while the rest is used for the ion pumps to maintain the cellular ion concentrations [19]. Cardiac energy is impaired in HF [10, 13, 18, 19, 53, 54]. Elevated catecholamines given to compensate for cardiac dysfunction in HF may do more harm than good and lead to a deterioration in cardiac bioenergetics [13, 55, 56]. Mitochondria are responsible for ATP production through oxidative phosphorylation. Several studies have shown that cardiac ETC is impaired and is accompanied with an increase in mitochondrial ROS generation in HF [20, 23, 24, 32, 57, 58]. Collectively, catecholamine has been shown to cause bioenergetics impairment in HF [10]. Our present study shows that continuous ISO stimulation results in cellular oxidative stress, cardiac remodeling, ETC impairment, mitochondrial superoxide elevation, cardiac bioenergetics alteration, and finally cardiac function deterioration.

Our present study shows that CAEA has anti-oxidative properties. CAEA recovered MnSOD expression and activity in mice subjected to ISO, subsequently alleviated cardiac oxidative stress, preserved cellular oxidative phosphorylation, cardiac energy, and cardiac function. The cardioprotective effect of CAEA was blocked by nicotinamide, inferring a sirtuin-dependent MnSOD restoration. This is in line with a previous study reporting a sirtuin-dependent MnSOD enhancement in AC5 knockout mice [59]. In view of the fact that some general antioxidants fail to treat HF, subcellular compartment signaling is believed to be the target for future drug development [26]. SIRT1 is found mostly in the nucleus and cytoplasm while SIRT3 is predominantly in the mitochondria [35, 36]. CAEA restored both SIRT1 and SIRT3 activity, and reduced cellular and mitochondrial oxidative stress. Hence, CAEA is a potential therapeutic candidate for preventing catecholamine-induced cardiac dysfunction during HF progression.

HIF-1α is a protein that regulates hypoxia-regulated gene expression to mediate cell adaption to low oxygen circumstances [60, 61]. ISO injections in rats have been shown to increase HIF-1α expression [62]. In the present study, ISO impaired cardiac ATP production while increasing the cardiac working load. This may have augmented HIF-1α expression due to relative hypoxia. HIF-1α is further stabilized by ROS or mitochondrial dysfunction [63, 64]. In addition, HIF-1α reprograms glucose metabolism from mitochondrial oxidative phosphorylation to glycolysis [65]. It has shown that metabolic remodeling in advanced HF includes elevated glycolysis, and a reduced respiratory chain activity [18]. This is consistent with the findings of the present study where ISO elevated cardiac HIF-1α expression, and the glycolysis rate. Hence, HIF-1α may be the missing link between oxidative stress and the metabolic shift seen in HF, resulting from chronic catecholamine stimulation.

CAEA reversed the HIF-1α elevation caused by ISO, which may result in the preservation of the cellular redox state. NAD^+^/NADH ratio represents the cellular redox state [18]. Through mitochondrial oxidative phosphorylation (OXPHOS), NADH produced by glycolysis is normally shuttled into the mitochondrial to generate ATP, H_2_O, CO_2_ and NAD^+^ that are shuttled back into the cytoplasm, maintaining the cellular and mitochondrial NAD^+^/NADH ratio [18]. Hence, mitochondrial OXPHOS is essential to maintain the cellular redox state. Studies have shown that HIF-1α increases anaerobic glycolysis accompanied with lactate accumulation. A prolonged lactate accumulation inhibits NAD^+^ regenerated from NADH, which leads to a decline in the NAD^+^/NADH ratio [18, 65]. In addition, a low NAD^+^/NADH ratio has been shown to enhance HIF-1α mediated mitochondrial OXPHOS inhibition that could cause a failure in the preservation of the cellular redox state [18, 66, 67]. These are compatible to the findings in the present study where CAEA restored mitochondrial OXPHOS, reduced HIF-1α expression and lactate content, and maintained cellular redox state in mice during chronic ISO treatment. Being NAD^+^-dependent deacetylases, the maintenance of sirtuins activity in the present study may be cooperatively by the preservation of intracellular NAD^+^.

### Study limitations

Our study does not provided a reason as to why CAEA was superior to caffeic acid in its cardioprotective effects on ISO-induced cardiac dysfunction. The mechanisms of the cardioprotective differences between CAEA and caffeic acid are planned for future studies.

## Conclusion

Our study shows that CAEA triggers intrinsic anti-oxidants in the cardiomyocyte, thus preventing oxidative stress-induced heart failure. CAEA also preserved the cardiac bioenergetic functions by oxidative phosphorylation restoration, HIF-1α expression reversal and cellular redox state maintenance. These findings suggest that the regulation of cardiac bioenergetics by SIRT1 and SIRT3 could increase heart tolerance to chronic stress and prevent catecholamine-induced cardiac dysfunction during HF progression.

## Abbreviations

CAEA: Caffeic acid ethanolamide
EF: Ejection fraction
ETC: Electric transport chain
HF: Heart failure
ISO: Isoproterenol
MnSOD: Manganese superoxide dismutase
OCR: Oxygen consumption rate
OXPHOS: Oxidative phosphorylation
ROS: Reactive oxygen species. ATP, Adenosine triphosphate
JNK: c-Jun N-terminal kinase
HIF-1α: Hypoxia-inducible factor 1-α
